# Evaluation of thyroid congestion in patients with heart failure using shear wave elastography: An observational study

**DOI:** 10.1097/MD.0000000000038159

**Published:** 2024-05-10

**Authors:** Takahiro Sakamoto, Toshihiko Asanuma, Kazuhiko Uchida, Hiroshi Kawahara, Akihiro Endo, Hiroyuki Yoshitomi, Kazuaki Tanabe

**Affiliations:** a Division of Cardiology, Shimane University Faculty of Medicine, Shimane, Japan; b Division of Cardiology, Masuda Red Cross Hospital, Shimane, Japan; c Department of Heart Failure and Transplant, National Cerebral and Cardiovascular Center, Osaka, Japan; d Department of Clinical Laboratory, Shimane University Hospital, Shimane, Japan

**Keywords:** hepatic congestion, organ congestion, right atrial pressure, shear wave elastography, ultrasonography

## Abstract

Shear wave elastography (SWE) is a noninvasive method for measuring organ stiffness. Liver stiffness measured using SWE reflects hepatic congestion in patients with heart failure (HF). However, little is known about the use of SWE to assess other organ congestions. This study aimed to evaluate the utility of SWE for assessing not only the liver but also thyroid congestion in patients with HF. This prospective study included 21 patients with HF who have normal thyroid lobes (age: 77.0 ± 11.0, men: 14). Thyroid and liver stiffness were measured by SWE using the ARIETTA 850 ultrasonography system (Fujifilm Ltd., Tokyo, Japan). SWE of the thyroid was performed on B-mode ultrasonography; a target region was identified within a region of interest. SWE was performed in each lobe of the thyroid gland. Five measurements were taken at the same location and the averages were recorded for comparison. We investigated the relationship between SWE for evaluating thyroid stiffness and the clinical characteristics of patients with HF. SWE of the thyroid was significantly correlated with SWE of the liver (*R* = 0.768, *P* < .001), thyroid stimulation hormone (*R* = 0.570, *P* = .011), free thyroxine (*R* = 0.493, *P* = .032), estimated right atrial pressure (RAP; *R* = 0.468, *P* = .033), and composite congestion score (*R* = 0.441, *P* = .045). SWE may be useful for evaluating thyroid stiffness and assessing the degree of thyroid congestion. Thyroid congestion may reflect the elevation of RAP and cause thyroid dysfunction through organ congestion.

## 1. Introduction

Heart failure (HF) causes congestion of various organs, including the liver, kidney, and intestine. Understanding organ congestion may help explain the interaction between the heart and other organs, such as that between the heart and kidney in cardio-renal syndrome, and a worsening function of either the heart or kidneys also affects other organs.^[1]^ Organ congestion can now be evaluated using extracardiac ultrasound, which is considered a novel technique.^[1]^ A recent study demonstrated that liver stiffness measured by shear wave elastography (SWE) using ultrasound is higher in patients with HF than in normal participants.^[2]^ Liver stiffness on elastography at admission and discharge has been found to reflect prognosis in patients with HF.^[3,4]^ However, little is known about measuring SWE to assess other organ congestions. This study aimed to evaluate the utility of SWE for assessing not only the liver but also thyroid congestion in patients with HF.

## 2. Methods

### 2.1. Patients and protocol

This prospective study included 21 patients with pre-HF or HF who were treated at the Masuda Red Cross Hospital between November and December 2018. HF was defined as a clinical syndrome of signs and/or symptoms caused by a structural and/or functional cardiac abnormality, which is corroborated by elevated natriuretic peptide levels and/or objective evidence of pulmonary or systemic congestion.^[5]^ Pre-HF was diagnosed in patients without current or prior signs and/or symptoms of HF but with evidence of structural heart disease, abnormal cardiac function, or elevated natriuretic peptide levels.^[5]^ No participant had a history or signs of thyroid and liver diseases, previous diagnosis of chronic thyroid and liver diseases, alcohol abuse (30 and 20 g/day for men and women, respectively), hepatic ultrasonography data indicating liver surface nodularity (a sign of severe fibrosis or ascites), anti-hepatitis C antibody positivity, or hepatitis B surface antigen reactivity. None of the participants were taking amiodarone.

SWE of the thyroid and liver, echocardiography, laboratory tests, and the composite congestion score (CCS) were collected on the same day. CCS was calculated by summing the individual scores (Table 1).^[6]^ The relationships between SWE of the thyroid and clinical characteristics of 21 patients with HF were investigated.

**Table 1:** Grading scale for investigator-assessed signs and symptoms of congestion.

Signs/symptoms	0	1	2	3
Dyspnea	None	Seldom	Frequent	Continuous
Orthopnea	None	Seldom	Frequent	Continuous
Fatigue	None	Seldom	Frequent	Continuous
JVD (cm H_2_O)	≤6	6 to 9	10 to 15	≥15
Rales	None	Bases	<50%	>50%
Edema	Absent/trace	Slight	Moderate	Marked

JVD = jugular venous distention.

The study protocol conformed to the principles outlined in the Declaration of Helsinki and was approved by the Masuda Red Cross Hospital Ethics Committee (approval number: 49). Informed consent was obtained from all the participants prior to their participation in the study.

### 2.2. Elastography

**Figure 1.**
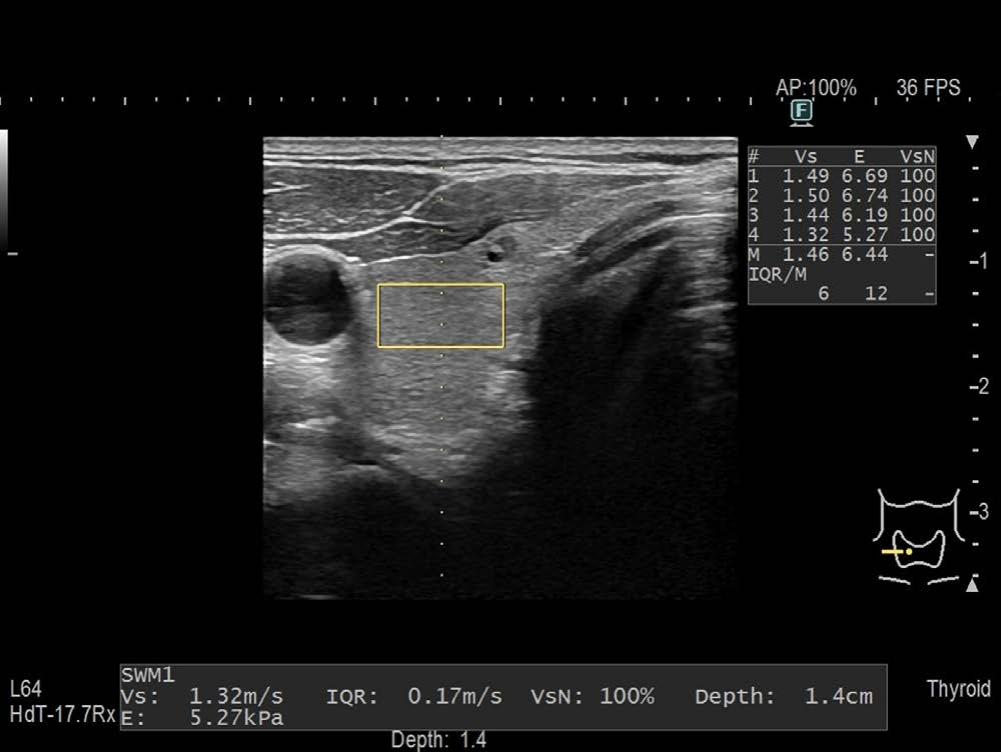
Identification of thyroid stiffness using shear wave elastography. Thyroid stiffness was determined using elastography. The shear wave velocity of the thyroid was measured using B-mode ultrasonography. A target region was identified within a region of interest as shown in the rectangle.

**Figure 2.**
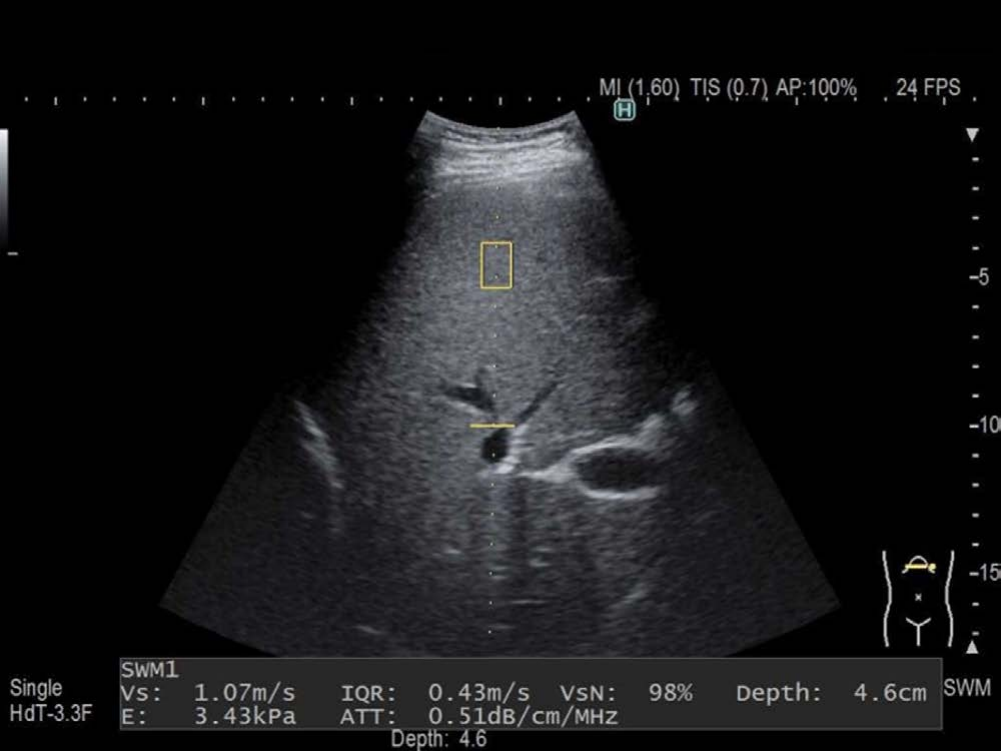
Identification of liver stiffness using shear wave elastography. A region of interest, devoid of large blood vessels, was located 1 to 2 cm below the liver surface.

A single experienced examiner (who had performed > 500 assessments), blinded to all clinical data, performed elastography using the commercially available ARIETTA 850 ultrasonography system (Fujifilm Ltd., Tokyo, Japan). Shear wave velocity (Vs) was measured using SWE, which is a method that calculates organ stiffness by measuring Vs shear waves propagate faster in harder substances than in softer ones. The equation “E = 3ρVs^2^” (E: elastic modulus [kPa]; ρ: density [kg/m^3^]) was applied based on the assumption that the body tissues were uniform. Therefore, tissue stiffness was calculated by measuring the propagation velocity of the shear waves. Vs of the thyroid was determined using B-mode ultrasonography as follows. First, a target region was identified within a region of interest (ROI). An acoustic push pulse was then transmitted with a shear wave generated within the target region. This shear wave was detected using sonographic detection pulses, and the numerical Vs values were displayed approximately within 2 seconds. Vs was measured in each lobe of the thyroid gland by pressing the probe lightly against the neck in the axial direction. The ROI was completely circumscribed by a thyroid lesion (Fig. 1). Sporea *et al* reported that 5 separate measurements are sufficient to assess thyroid stiffness; therefore, 5 measurements were performed at the same location, and the average was recorded for comparison.^[7,8]^ SWE of the liver was performed in the right intercostal space, inferior to the right anterior axillary line, with the patient in a supine position and the right arm maximally abducted from the liver. An ROI, devoid of large blood vessels, was located 1 to 2 cm below the liver surface (Fig. 2). A successful recording of Vs was defined as ≥ 5 successful readings with a net effective Vs ≥ 50%.^[9]^ Training was needed for clear visualization of the thyroid and liver by ultrasound imaging and performing SWE in an area without artifacts.

### 2.3. Laboratory tests and echocardiography

On-site laboratory tests, including routine tests such as the liver function and brain natriuretic peptide tests, were performed on the same day of performing SWE. Echocardiography was performed by experienced sonographers, as per the recommendations of the American Society of Echocardiography and the European Association of Cardiovascular Imaging.^[10]^ Left ventricular ejection function (LVEF) was calculated from the apical 4- and 2-chamber views using the biplane method of disks. Similarly, the left atrial volume was measured from standard apical 2- and 4-chamber views at end-systole. The left atrial volume index was calculated by dividing the left atrial volume by body surface area. Peak early diastolic (E) and late diastolic velocities (A) were measured from transmitral flow velocity curves, whereas early diastolic myocardial velocities (e’) were obtained from tissue Doppler imaging of the mitral annulus at the septal position. The left ventricular outflow tract velocity-time integral was calculated by placing the pulsed Doppler sample volume in the outflow tract below the aortic valve and recording the velocity. From the subcostal view, the inferior vena cava (IVC) diameter was measured within 3 cm of the right atrium–IVC junction during passive respiration. The estimated right atrial pressure (RAP) was determined based on the IVC diameter and collapse with a sniff.^[11]^ In addition, tricuspid regurgitation was graded on a 4-point scale based on color-flow Doppler images.

### 2.4. Statistical analysis

Continuous variables are expressed as median (interquartile range). Categorical variables are expressed as numbers and percentages of patients. Relationships between elastography of the thyroid and other variables were assessed by Pearson correlation analyses for continuous variables or by Spearman rank correlation analysis for discrete variables. All statistical analyses were performed using EZR, version 1.54 (Saitama Medical Center, Jichi Medical University, Saitama, Japan), a graphical user interface for R, version 4.0.3 (The R Foundation for Statistical Computing, Vienna, Austria).^[12]^ A 2-sided *P*-value of ≤ 0.05 was considered statistically significant.

## 3. Results

### 3.1. Baseline characteristics

The baseline characteristics of the participants are shown in Table 2. The laboratory data, echocardiography findings, and CCS are summarized in Table 3. The median age of patients with HF was 80 years. Among them, 13 (62%) were males, and 4 (19%) had New York Heart Association functional class III/IV. The underlying causes of HF were valvular heart disease (8 patients, 38%), cardiomyopathy (6 patients, 29%), ischemic heart disease (2 patients, 10%), and other conditions (5 patients, 24%). The median BNP level was 494 pg/mL, and the LVEF was 60%. Free triiodothyronine and free thyroxine levels were within normal ranges.

**Table 2:** Baseline characteristics of the participants.

	Patients (n = 21)
Age, years	80 (71–86)
Male, n (%)	13 (62)
Body mass index, kg/m^2^	22 (19–24)
Systolic blood pressure, mm Hg	130 (103–145)
Heart rate, bpm	74 (68–85)
New York Heart Association class III/IV, n (%)	4 (19)
Medical history, n (%)	
Hypertension	20 (95)
Diabetes mellitus	6 (29)
Dyslipidemia	11 (52)
Chronic kidney disease	15 (71)
Atrial fibrillation	6 (29)
HF etiology, n (%)	
Ischemia	2 (10)
Valvular heart disease	8 (38)
Cardiomyopathy	6 (29)
Others	5 (24)
Medications, n (%)	
ACEI or ARB	13 (62)
β-blocker	11 (52)
Mineral corticoid-receptor antagonists	7 (33)
Diuretics	10 (48)

Values are expressed as median (interquartile range) or number (%). ACEI = angiotensin-converting enzyme inhibitor, ARB = angiotensin receptor blocker, HF = heart failure

**Table 3:** Laboratory data, echocardiography findings, and composite congestion score.

	Patients (n = 21)
Laboratory data	
Hemoglobin, g/dL	12 (10–13)
Platelet, ×10^3^/μL	20 (18–24)
T-Bil, mg/dL	0.6 (0.5–0.8)
AST, U/L	25 (18–31)
ALT, U/L	17 (12–28)
γ-GTP, U/L	19 (15–74)
ALP, U/L	251 (201–279)
eGFR, mL/min/1.73 m^2^	33 (27–55)
BNP, pg/mL	494 (72–593)
TSH, IU/mL	2.3 (1.5–3.6)
Free triiodothyronine, pg/mL	2.6 (2.3–3.0)
Free thyroxine, ng/dL	1.1 (0.9–1.2)
Echocardiography	
LVEDV, mL	88 (66–102)
LVEF, %	60 (50–70)
LAVI, mL/m^2^	48 (33–55)
E/A	0.8 (0.7–0.9)
E/e’	13 (10–17)
LVOT-VTI, cm	20 (18–23)
TRPG, mm Hg	31 (21–35)
Maximal IVC diameter, mm	13 (11–15)
TR III/IV, n (%)	3 (14)
CCS	1 (0–3)

Values are expressed as median (interquartile range) or number (%). A = late transmitral flow velocity, ALP = alkaline phosphatase, ALT = alanine aminotransferase, AST = aspartate aminotransferase, BNP = brain natriuretic peptide, CCS = composite congestion score, E = early transmitral flow velocity, E/e’ = ratio of peak mitral E wave velocity to peak early diastolic myocardial velocity at the septal and lateral positions recorded using tissue Doppler imaging, eGFR = estimated glomerular filtration rate, IVC = inferior vena cava, LAVI = left atrial volume index, LVEDV = left ventricular end-diastolic volume, LVESV = left ventricular end-systolic volume, LVEF = left ventricular ejection fraction, LVOT-VTI = left ventricular outflow tract velocity-time integral, T-Bil = total bilirubin, TR = tricuspid regurgitation, TRPG = tricuspid regurgitation peak gradient, TSH = thyroid stimulation hormone, γ-GTP = gamma-glutamyl transpeptidase.

### 3.2. SWE of the thyroid in patients with HF

**Figure 3.**
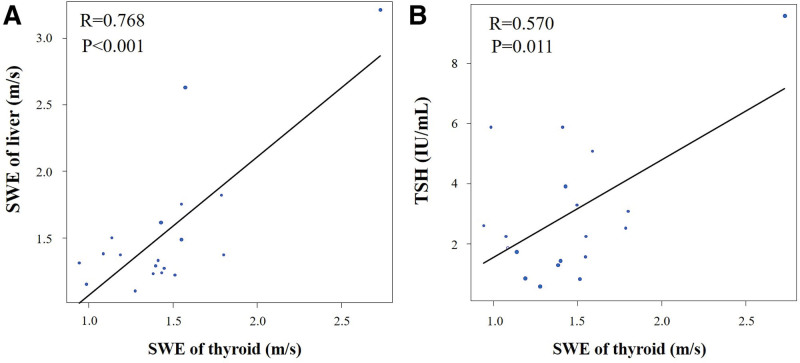
Scatter plot diagrams showing the correlation of shear wave elastography (SWE) of the thyroid with (A) SWE of the liver and (B) thyroid stimulation hormone (TSH). SWE of the thyroid was significantly correlated with SWE of the liver and TSH.

SWE of the thyroid in patients with HF was significantly correlated with SWE of the liver (*R* = 0.768, *P* < .001), thyroid stimulation hormone (TSH; *R* = 0.570, *P* = .011), free thyroxine (*R* = 0.493, *P* = .032), LVEF (r = –0.593, *P* = .005), E/A (r = –0.622, *P* = .010), E/e’ (*R* = 0.484, *P* = .049), estimated RAP (*R* = 0.468, *P* = .033), and CCS (*R* = 0.441, *P* = .045) (Figure 3, Table 4).

**Table 4:** Relationship between elastography of the thyroid and clinical characteristics.

	Elastography for thyroid	
	*R*	*P* value
Laboratory data		
Hemoglobin, g/dL	−0.163	0.480
Platelet, ×10^3^/μL	−0.380	0.090
T-Bil, mg/dL	0.090	0.698
AST, U/L	0.234	0.321
ALT, U/L	−0.020	0.928
γ-GTP, U/L	0.343	0.150
ALP, U/L	0.046	0.852
eGFR, mL/min/1.73 m^2^	−0.243	0.348
BNP, pg/mL	0.319	0.158
Free triiodothyronine, pg/mL	−0.167	0.552
Free thyroxine, ng/dL	0.493	0.032
Echocardiography		
LVEDV, mL	0.282	0.216
LVEF, %	−0.593	0.005
LAVI, mL/m^2^	0.063	0.788
E/A	0.622	0.010
E/e’	0.484	0.049
LVOT-VTI, cm	−0.361	0.205
TRPG, mm Hg	−0.138	0.574
Estimated RAP, mm Hg	0.468	0.032
CCS	0.441	0.045

A = late transmitral flow velocity, ALP = alkaline phosphatase, ALT = alanine aminotransferase, AST = aspartate aminotransferase, BNP = brain natriuretic peptide, CCS = composite congestion score, E = early transmitral flow velocity, E/e’ = ratio of peak mitral E wave velocity to peak early diastolic myocardial velocity at the septal and lateral positions recorded using tissue Doppler imaging, eGFR = estimated glomerular filtration rate, LAVI = left atrial volume index, LVEDV = left ventricular end-diastolic volume, LVEF = left ventricular ejection fraction, LVOT-VTI = left ventricular outflow tract velocity-time integral, RAP = right atrial pressure, T-Bil = total bilirubin, TRPG = tricuspid regurgitation peak gradient, TSH = thyroid stimulation hormone, γ-GTP = gamma-glutamyl transpeptidase.

## 4. Discussion

In this study, SWE of the thyroid in patients with HF was significantly correlated with estimated RAP, CCS, SWE of the liver and TSH level. To the best of our knowledge, this is the first study to demonstrate thyroid congestion using SWE in patients with HF.

### 4.1. Thyroid congestion

It has been increasingly recognized that end-organ damage, such as hepatic and renal dysfunction, increases due to venous congestion in patients with HF.^[13]^ The thyroid gland is a butterfly-shaped endocrine organ consisting of the right and left lateral lobes and isthmus. The right and left superior, middle, and inferior thyroid veins are observed. The superior and middle thyroid veins join the internal jugular vein, the right inferior thyroid vein enters the brachiocephalic vein, and the left inferior thyroid vein enters the subclavian vein, all of which join the superior vena cava. In this study, SWE of the thyroid was correlated with the estimated RAP. This suggests that elevated RAP caused thyroid congestion via the superior vena cava, resulting in high Vs values.

SWE of the thyroid was also correlated with SWE of the liver. Since liver stiffness reflects hepatic congestion in patients with HF, our results indicate that patients with hepatic congestion may also have thyroid congestion.^[14]^ A previous study has reported that elevated RAP causes thyroid dysfunction through thyroid congestion.^[15]^ Elevated central venous pressure reflects intravascular congestion, whereas organ congestion reflects tissue congestion. Although many cases present with both, it has been suggested that an alternative diagnostic approach may be required.^[16]^ Evaluation of direct tissue congestion via thyroid elastography, rather than the traditional estimation of RAP using echocardiographic indices, may help to further clarify the relationship between thyroid congestion and abnormal thyroid function.

### 4.2. Subclinical hypothyroidism due to thyroid congestion

Subclinical hypothyroidism is associated with cardiovascular adverse events such as atherosclerosis, coronary artery disease, and increased risk of HF.^[17–19]^ In patients with HF, the higher the TSH level, the poorer the prognosis.^[19]^ In addition, thyroid congestion has been reported to be associated with subclinical hypothyroidism in patients with a Fontan circulation.^[15]^ A previous study has reported that TSH levels are significantly correlated with RAP.^[15]^ Considering that high TSH levels may reflect thyroid congestion in patients with HF, and residual congestion may be associated with a poorer prognosis.

In this study, SWE of the thyroid was found to be positively correlated with both free thyroxine and TSH. However, free thyroxine was within the normal range in all of the cases, while TSH was elevated to the point of subclinical hypothyroidism in some (TSH ≥ 5 IU/mL). Since TSH is thought to reflect hypothyroidism earlier and more accurately than free thyroxine, thyroid congestion may have been the cause of this subclinical hypothyroidism in our patient cohort. Further studies are required to elucidate this cardio-thyroid relationship.

### 4.3. Limitations

This study has some limitations. First, the sample size was small. Second, we were unable to compare the elastography findings at admission and discharge. Third, there was no comparison between elastography and central venous pressure measured by right heart catheterization. The RAP was estimated using echocardiography. The reliance on noninvasive estimation represents a significant limitation of this study. Fourth, this study did not investigate clinical outcomes, which weakens its clinical implications. Finally, thyroid antibodies were not measured; therefore, the possibility of a thyroid disease could not be ruled out. However, because free triiodothyronine and free thyroxine levels were normal in all patients, the clinical significance of measuring thyroid antibodies would have been low.

### 4.4. Conclusions

SWE is useful for evaluating thyroid stiffness and assessing the degree of thyroid congestion. Thyroid congestion may reflect the elevation of RAP and cause subclinical hypothyroidism through organ congestion.

## Author contributions

**Conceptualization:** Takahiro Sakamoto.

**Data curation:** Takahiro Sakamoto.

**Formal analysis:** Takahiro Sakamoto.

**Investigation:** Takahiro Sakamoto.

**Methodology:** Takahiro Sakamoto.

**Project administration:** Takahiro Sakamoto.

**Resources:** Takahiro Sakamoto.

**Software:** Takahiro Sakamoto.

**Validation:** Takahiro Sakamoto.

**Visualization:** Takahiro Sakamoto.

**Writing – original draft:** Takahiro Sakamoto.

**Writing – review & editing:** Takahiro Sakamoto, Toshihiko Asanuma, Akihiro Endo, Kazuaki Tanabe.

**Supervision:** Toshihiko Asanuma, Kazuhiko Uchida, Hiroshi Kawahara, Akihiro Endo, Hiroyuki Yoshitomi, Kazuaki Tanabe.
